# Cost‐effectiveness analysis of abrocitinib compared with standard of care in adult moderate‐to‐severe atopic dermatitis in Japan

**DOI:** 10.1111/1346-8138.17234

**Published:** 2024-04-22

**Authors:** Akio Tanaka, Akira Yuasa, Kazumasa Kamei, Mitsuhiro Nagano, Toshiaki Murofushi, Annika Bjerke, Kouki Nakamura, Shunya Ikeda

**Affiliations:** ^1^ Department of Dermatology, Graduate School of Biomedical and Health Sciences Hiroshima University Hiroshima Japan; ^2^ Japan Access & Value Pfizer Japan Inc. Tokyo Japan; ^3^ HEOR, Value & Access INTAGE Healthcare Inc. Tokyo Japan; ^4^ Modeling and Analytics Lumanity Bethesda Maryland USA; ^5^ Medical Affairs Pfizer Japan Inc. Tokyo Japan; ^6^ Department of Public Health, School of Medicine International University of Health and Welfare Narita Chiba Japan

**Keywords:** abrocitinib, atopic dermatitis, cost‐effectiveness analysis, productivity loss, Japan

## Abstract

Atopic dermatitis (AD) is a chronic inflammatory skin disease with a significant clinical, economic, and human burden. The JAK1 Atopic Dermatitis Efficacy and Safety (JADE) program's Phase 3 trials demonstrated that as a treatment for moderate‐to‐severe AD in adults with previous exposure to immunotherapy, abrocitinib showed superior efficacy and safety compared with standard of care (SoC), consisting of topical corticosteroids. This study assessed the cost‐effectiveness of abrocitinib with SoC versus SoC alone for this patient population in Japan from a societal perspective. A hybrid decision tree and Markov model were used to capture the initial treatment and long‐term maintenance phases. Clinical inputs at 16 weeks were obtained through a Bayesian network meta‐analysis of four pivotal trials from the JADE program. Clinical inputs at 52 weeks were derived from the JADE EXTEND trial. Response‐specific utility inputs were obtained from published literature. Resource use, costs, and productivity inputs were gathered from Japanese claims analysis, literature, public documents, and expert opinion. Costs and quality‐adjusted life years (QALYs) were discounted at 2.0% per year and incremental cost‐effectiveness ratios (ICERs) were calculated. Sensitivity and scenario analyses were performed to validate the base case results and explore a payer perspective. Over a lifetime horizon and with the base‐case societal perspective, abrocitinib produced a mean gain of 0.75 QALYs, incremental costs of JPY (¥) 2 270 386 (USD [$] 17 265.6), and a resulting ICER of ¥3 034 514 ($23 076.5) per QALY compared with SoC. From a payer perspective, the incremental costs increased to ¥4 476 777 ($34 044.4), with an ICER of ¥5 983 495 ($45 502.6) per QALY. The results were most sensitive to treatment‐specific, response‐based utility weights, drug costs, and productivity‐related inputs. From a Japanese societal perspective, abrocitinib demonstrated superior QALYs and with a willingness‐to‐pay threshold of ¥5 000 000 ($38 023.4) per QALY, can be considered cost‐effective compared with SoC as a treatment for moderate‐to‐severe AD in adult patients with previous immunosuppressant exposure.

## 1 INTRODUCTION

Atopic dermatitis (AD) is a chronic inflammatory skin disease that is characterized by dry skin and red itchy lesions that can occur anywhere on the body in a persistent or relapsing manner.^1^ AD affects both children and adults, although 60% of cases begin during childhood.^2^ The prevalence of AD has increased by an estimated 10%–20% in developed countries over the last 30 years.^2^ In Japan, AD affects approximately 10% of the population among people of all ages.^3^ Patients are diagnosed with moderate AD when eruptions with severe inflammation are observed on <10% of the body surface area, and with severe AD when eruptions with severe inflammation are observed on >10% to <30% of the body surface area.^4^, ^5^ AD has a considerable impact on a patient's quality of life, including their sleep, psychosocial well‐being, and ability to engage in everyday activities, and it presents a high economic burden to society.^6^, ^7^, ^8^ In fact, a recent study estimated the cost of illness for adult AD patients in Japan to be approximately 3 trillion JPY (¥) annually.^6^

Over the last few years, several new drugs have been approved in Japan to treat moderate‐to‐severe AD. Dupilumab, an anti‐interleukin (IL)‐4 receptor α monoclonal antibody, was the first systemic treatment approved in 2018.^9^, ^10^ The authorized indications for baricitinib, an oral Janus kinase inhibitor (JAKi), were expanded to include AD in 2020.^11^ In 2021, another oral JAKi, upadacitinib, was approved to treat AD, followed shortly thereafter by abrocitinib.^11^

Abrocitinib, a JAK1‐selective inhibitor, is the latest JAKi to be approved by the Japanese Ministry of Health, Labour and Welfare (MHLW).^12^ In Japan, abrocitinib was approved for the treatment of moderate‐to‐severe AD in adults and adolescents aged ≥12 years who showed an inadequate response to existing therapies. Abrocitinib is available in doses of 100 and 200 mg.^12^ The standard dose for abrocitinib is 100 mg.^13^

Several cytokines, such as IL‐4, IL‐13, IL‐22, IL‐31, thymic stromal lymphopoietin, and interferon‐γ, are involved in the pathogenesis of AD.^14^, ^15^, ^16^ As oral JAKi hinder the JAK‐signal transducers and activators of transcription signal transduction routes involved in the signal transduction of these cytokines, they may be effective in treating AD.^17^, ^18^, ^19^

Approval of the drug in all countries was based on trial results from several Phase 3 studies and a related long‐term extension study. In Japan, the most recent guidance for the use of oral JAKi in the treatment of AD recommended abrocitinib based on the JAK1 Atopic Dermatitis Efficacy and Safety (JADE) COMPARE (NCT037204700), JADE TEEN (NCT03796676), and JADE Mono‐2 (NCT03575871) trial results.^11^

As Japan is facing increasing medical expenditure caused by high drug prices, a cost‐effectiveness analysis (CEA) is critical, to manage drug expenditure as well as assess the value for money of introducing a new medical intervention to the healthcare system. CEAs are particularly useful in their ability to provide insight into indirect costs, such as productivity losses, which are especially notable in AD.^6^, ^20^

Productivity losses are often captured through overall work impairment (OWI), which considers both absenteeism and presenteeism in employed AD patients using a Work Productivity and Activity Impairment Questionnaire (WPAI).^6^, ^7^ Within the Japanese context, several studies have estimated that the OWI of employed adults with AD in Japan is approximately 30%–34%.^6^, ^7^, ^8^ All three studies have noted that the majority of the OWI comprises presenteeism rather than absenteeism.^6^, ^7^, ^8^

Despite the availability of several health technology assessment (HTA) submissions on AD,^21^, ^22^, ^23^, ^24^, ^25^, ^26^ only a few of these are specific to abrocitinib and none focus on the Japanese context. Additionally, no CEAs have adopted a societal perspective in a base‐case setting.^21^, ^25^ However, several CEAs have considered productivity loss in a scenario analysis using a wider societal perspective.^21^, ^22^

Given, the potential impact of productivity loss on analysis outcomes, including total costs and incremental cost‐effectiveness ratios (ICERs), it is an important and relevant to include productivity losses when performing a CEA for AD. Consequently, this CEA assessed the cost‐effectiveness of abrocitinib with standard of care (SoC) versus SoC alone in adults with moderate‐to‐severe AD in Japan using a societal perspective as the base case.

## 2 METHODS

### 2.1 Patient population, perspective, intervention, and comparators

The patient population of the model consisted of adults aged ≥18 years with moderate‐to‐severe AD and previous exposure to immunosuppressants who were eligible for systemic therapy.

To align with Japanese clinical practice, abrocitinib 100 mg was the chosen dose for the analysis and was used in combination with low‐ or medium‐potency topical corticosteroids (TCS), topical calcineurin inhibitors, or topical phosphodiesterase‐4 inhibitors as the intervention. The only comparator included in the analysis was SoC, consisting of low‐ or medium‐potency TCS, topical calcineurin inhibitors, or topical phosphodiesterase‐4 inhibitors. SoC was selected based on the Japanese guideline for cost‐effectiveness evaluation,^27^ which notes that the comparator should be principally selected from drugs that are widely used in clinical practice and are expected to be replaced by the intervention of interest when it is introduced to treat the target population.

The base‐case analysis of this study was conducted from a Japanese societal perspective, which considered costs associated with productivity loss of patients due to AD treatment.^28^ A Japanese payer perspective was considered in the scenario analysis. The main distinction between the base case and scenario settings is that the Japanese societal perspective considers both direct healthcare costs and productivity loss, while the Japanese payer perspective considers only direct healthcare costs.

### 2.2 Model structure and settings

**FIGURE 1:**
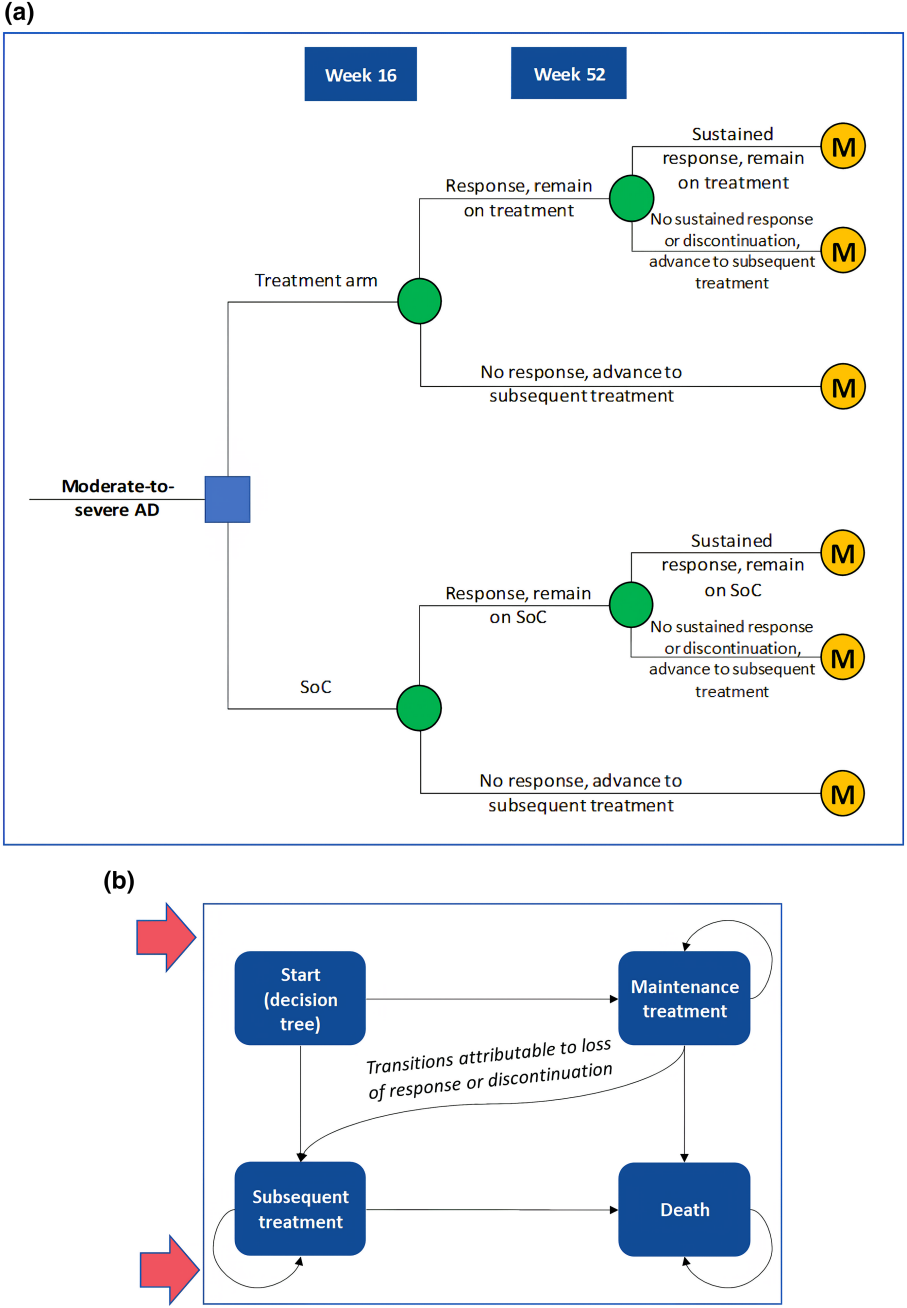
Model structure diagram. (a) Decision tree: initial phase of treatment, which captures patients from treatment initiation up to 52 weeks. (b) Markov model: maintenance phase, which captures patients after 52 weeks. AD, atopic dermatitis; M, Markov model; SoC, standard of care.

The model, developed in Microsoft Excel®, was structured as a 52‐week decision tree, followed by a three‐state Markov model. The hybrid nature of the model, shown in Figure 1, was chosen to capture the initial and maintenance phases of treatment with abrocitinib. In this study, the same analysis model as the one used for the HTA submission of abrocitinib and dupilumab in Canada was used.^21^, ^29^ It was judged to be fit for the decision‐making purpose by the Canadian HTA agency as the analysis model properly reflected the actual treatment of AD in clinical practice.

Decision‐tree model analysis involves identifying the expected costs and effects of following a patient through the clinical pathways resulting from a clinical decision. Markov model analysis is a method whereby cost‐effectiveness is considered based on the probability of transitioning patients from one health state to another. Patients cycle through (in other words, move through in a probabilistic way) predefined health states relevant to the disease being evaluated and accumulate costs and effects based on the state they occupy. AD is often treated with pharmacological medications that may be discontinued after a certain period if the patient's condition improves. However, some patients require long‐term treatment. To model the costs and outcomes of both the initial treatment phase and maintenance treatment, a hybrid decision model was used.

The first year of the model, structured as a decision tree, captured the initial phase of treatment where patients received either abrocitinib with TCS or SoC (i.e., TCS alone). Patient response to treatment was evaluated at 16 weeks and re‐evaluated at 52 weeks. Patients who received abrocitinib and did not demonstrate a sufficient response at either time point or discontinued for any reason transitioned to SoC, while responding patients moved on to receive maintenance treatment. In the decision‐tree phase of the model, patients accrued costs and quality‐adjusted life years (QALYs) dependent on their treatment response status (responder or non‐responder).

The Markov state transition model captures the maintenance phase. Should patients demonstrate response to abrocitinib at 16 weeks, continue treatment, and present a sustained response up until 52 weeks, they would continue to receive abrocitinib as a maintenance therapy until (a) they experience loss of response and/or discontinue, whereby they transition to SoC, or (b) they die. The transition to SoC due to loss of response and discontinuation was modeled as a constant linear rate of discontinuation. The per‐cycle rate was derived using the abrocitinib discontinuation data at 52 weeks based on the JADE EXTEND (NCT03422822) trial. The model assumed that patients did not discontinue from SoC.

The model time horizon was a lifetime horizon to capture all time relevant costs and efficacy of the treatments analyzed. The model cycle length was 6 months, with half‐cycle correction implemented.

The model outcomes were costs and QALYs, discounted at an annual rate of 2.0%, which is a standard setup for cost‐effectiveness analysis conducted in Japan in accordance with a public guideline.^27^ They were used to calculate ICERs versus SoC.

A willingness‐to‐pay (WTP) threshold of ¥ 5 000 000 (USD [$] 38 023.4) per QALY was used for the analysis.^31^

### 2.3 Effectiveness and safety

Effectiveness and safety outcomes considered in this analysis included measures of disease severity, measures of symptom control, disease‐free period/maintenance of remission, time to relapse/prevention of relapse, and adverse effects of treatment.

Clinical comparative efficacy inputs at 16 weeks for abrocitinib and SoC were derived from a Bayesian network meta‐analysis (NMA) that considered three of the four abrocitinib Phase 3 trials (JADE Mono‐1 [NCT03349060], Mono‐2, and COMPARE), along with other relevant studies for the NMA.^32^ The outcome for defining responders from the NMA used as the base case in this analysis was the ordered multinomial composite outcome, the Eczema Area and Severity Index (EASI) score of 75 with a ≥4‐point improvement in the Dermatology Life Quality Index (DLQI) from baseline, using odds ratios to measure the relative efficacy. In other words, in the base case of this analysis, patients were considered responders if they achieved ≥75% improvement from baseline with respect to the EASI score, with a ≥ 4‐point improvement with respect to DLQI. This outcome was taken at 16 weeks for the JADE COMPARE trial and at 12 weeks for the JADE Mono‐1 and Mono‐2 trials. Given abrocitinib's fast onset of action, no notable differences in efficacy would be expected between 12 and 16 weeks, as was evidenced in the JADE COMPARE trial.^33^ Clinical inputs at 52 weeks (i.e., sustained response at 52 weeks among responders at 16 weeks) were derived from the JADE EXTEND clinical trial for abrocitinib and SoC.

Based on the NMA results, at 16 weeks, 42.9% and 10.7% of patients that received abrocitinib and SoC were responders, respectively. For long‐term responders, 80.6% and 72.5% of patients that received abrocitinib and SoC would experience a sustained response at 52 weeks, respectively. A linear waning of long‐term treatment response occurring over 10 years for abrocitinib and over 5 years for SoC was assumed and response rates at 10 years (abrocitinib) and 5 years (SoC) were 62.0%^34^ (assumed to be the same as dupilumab long‐term data) and 3.0%,^29^ respectively.

In the base case, approximately 6.9% of patients receiving abrocitinib were assumed to discontinue between 16 and 52 weeks.^34^ The discontinuation rate following 52 weeks was assumed to be the same as dupilumab (6.3% annually).^29^ For SoC, no discontinuation was assumed.

As AD is not expected to directly affect survival, it was assumed that there was no treatment effect on mortality, and as such, survival was the same for both treatment arms. The latest Japanese Life Table was used for modeling survival.^35^

Frequency of adverse events (all‐cause Grade 3+ adverse events ≥5%) were based on the pooled analyses of the JADE trials for abrocitinib. Of note, herpes zoster is identified as a specific adverse event of interest in Japan related to treatment with JAKi^36^ and was included in the analysis regardless of the cut‐off threshold.

### 2.4 Utility

Utility values were dependent on responder/non‐responder status and on whether patients received abrocitinib or SoC. Adverse event disutilities were not considered, which was consistent with past dupilumab HTA assessments in AD.^29^ In the base case, utility weights were derived from published literature,^37^ based on EuroQol 5 dimensions 3‐level (EQ‐5D‐3L) questionnaire results collected from the clinical trials of dupilumab including SOLO 1 (NCT02277743) and SOLO 2 (NCT02277769). While a post‐hoc analysis was conducted using EuroQol 5 dimensions 5‐level (EQ‐5D‐5L) data collected from JADE COMPARE and EXTEND trials and EQ‐5D‐5L data collected from JADE Mono‐1 and Mono‐2, both of which were mapped to EQ‐5D‐3L, the dupilumab trial values were considered the most appropriate as the JADE trials excluded patients with clinically significant depression, which substantially affected the utility values derived from the abrocitinib studies.

### 2.5 Costs

Drug acquisition and healthcare resource utilization (HCRU) costs were valued in JPY at the time of utilization, and the most recent Medical Service Fee Points in Japanese National Health Insurance (NHI) data were used where applicable.^38^ Additionally, JPY were adjusted to USD using the annual exchange rate in 2022 (Organization for Economic Co‐operation and Development, $ 1 = ¥ 131.498).^39^ Costs were calculated by multiplying frequency of HCRU and unit cost. Frequency of HCRU was estimated using the Japanese claims database provided by Medical Data Vision Co., Ltd., which captures data from over 40 million patients who have attended Japanese public and private healthcare facilities since 2013.^40^ The unit cost of each drug was taken from the list price according to NHI data (revised in 2024).^41^ Drug acquisition costs were based on drug unit costs, dosing schedules, and time on treatment. Drug unit costs were ¥4287.40 ($32.6) for 100 mg of abrocitinib, while SoC drug costs were assumed to be ¥0.00 ($0.0) as TCS was a component of both treatment arms. Administration costs were not considered as neither abrocitinib nor SoC include injectable treatments.

This analysis considered disease management and monitoring costs using a micro‐costing approach. The frequency at which patients required hospitalization and primary care visits was dependent on response status. For responders, total annual HCRU costs were ¥148 959 ($1132.8), while for non‐responders these were ¥219 175 ($1666.8). Monitoring costs captured laboratory testing and were separately modeled for Year 1 and Year 2 onwards by treatment received. It was assumed that patients who received SoC would not require monitoring tests, while for patients who received abrocitinib, their annual testing costs were estimated at ¥13 270 ($100.9). The frequency of laboratory testing was validated by clinical experts, Akio Tanaka (Department of Dermatology, Graduate School of Biomedical and Health Sciences, Hiroshima University) and Kouki Nakamura (Pfizer Japan Inc.), and the costs were derived from NHI Medical Service Fee Points.^38^ Adverse event management costs ranged from ¥730 ($5.6) to ¥4776 ($36.3) and were assumed to be equivalent to the cost of a subsequent visit and drugs used to manage each adverse event. Subsequent treatment costs were applied monthly and sourced from the literature.^6^

For the base‐case societal perspective, the productivity‐related inputs were derived from Japanese government statistics^42^, ^43^, ^44^ and the study by Murota et al.^6^ The retirement age was assumed to be 65 years. The estimated proportion of working hours lost at baseline for patients with moderate‐to‐severe AD and the reduction of lost working hours for responders were based on the report by Murota et al.^6^ It was assumed that non‐responders had the same number of lost working hours as those at baseline. Key model inputs are summarized in Table 1.

**TABLE 1:** Key base‐case model input parameters.

Parameters	Inputs		References
Drug acquisition cost (abrocitinib)	¥4287.40 per 100 mg pill		NHI drug price list (revised in 2024)^41^
Medical resource utilization unit costs			
Hospitalization, mean cost per visit (SE)	¥695 973.3 (¥5719.7)		Estimation based on Medical Data Vision Co., Ltd claims analysis^40^ and NHI Medical Service Fee Points^38^
Primary care visits, mean cost per visit (SE)	¥7096.8 (¥19.9)		
Monitoring test unit costs			
Renal function test	¥110		NHI Medical Service Fee Points (D007)^38^
Complete blood count	¥210		NHI Medical Service Fee Points (D005)^38^
Liver function test	¥170		NHI Medical Service Fee Points (D007)^38^
Tuberculosis testing	¥6100		NHI Medical Service Fee Points (D015, D291)^38^
Hepatitis B and C testing	¥3600		NHI Medical Service Fee Points (D013)^38^
Chest X‐ray	¥2100		NHI Medical Service Fee Points (E001 and E002)^38^
Adverse event management cost per event			
Acne	¥4776		Validated by clinical experts based on NHI list price^38^ and NHI drug price list (revised in 2024)^41^
Conjunctivitis	¥1178		Validated by clinical experts based on NHI list price^38^ and NHI drug price list (revised in 2024)^41^
Dermatitis Atopic	¥1114		Validated by clinical experts based on NHI list price^38^ and NHI drug price list (revised in 2024)^41^
Headache	¥730		Validated by clinical experts based on NHI list price^38^
Injection site reaction	¥730		Validated by clinical experts based on NHI list price^38^
Nasopharyngitis	¥730		Validated by clinical experts based on NHI list price^38^
Nausea	¥730		Validated by clinical experts based on NHI list price^38^
Oral herpes	¥1173		Validated by clinical experts based on NHI list price^38^ and NHI drug price list (revised in 2024)^41^
Sinusitis	¥730		Validated by clinical experts based on NHI list price^38^
Upper respiratory tract infection	¥730		Validated by clinical experts based on NHI list price^38^
Urinary tract infection	¥841		Validated by clinical experts based on NHI list price^38^ and NHI drug price list (revised in 2024)^41^
Vomiting	¥730		Validated by clinical experts based on NHI list price^38^
Folliculitis	¥859		Validated by clinical experts based on NHI list price^38^ and NHI drug price list (revised in 2024)^41^
Herpes zoster	¥2591		Validated by clinical experts based on NHI list price^38^ and NHI drug price list (revised in 2024)^41^
Subsequent treatment cost per month			
Subsequent treatment following abrocitinib	¥14 451		Murota et al.^6^
Subsequent treatment following SoC	¥14 451		
Response rates at *n* weeks, by treatment, %			
Abrocitinib, 16 weeks	42.9		NMA^32^ (incl. JADE Mono‐1 [NCT03349060], Mono‐2 [NCT03575871], COMPARE [NCT037204700])
SoC, 16 weeks	10.7		
Abrocitinib, 52 weeks	80.6		JADE EXTEND (NCT03422822)
SoC, 52 weeks	72.5		
Duration of long‐term response			
Abrocitinib	5 years		Assumption
SoC	10 years		Assumption
Long‐term response rates,%			
Abrocitinib	62.0		Assumed equal to dupilumab^29^
SoC	3.0		CADTH report^29^
Discontinuation rates, by treatment at week(s) n, %			
Abrocitinib, Week 16–52	6.9		NMA^32^ (incl. JADE Mono‐1 [NCT03349060], Mono‐2 [NCT03575871], COMPARE [NCT037204700])
SoC, Week 16–52	0.0		Assumption
Abrocitinib, Week 52+	6.3		Assumed equal to dupilumab^29^
SoC, Week 52+	0.0		Assumption
Disease management resource frequency of use per			
Hospitalization annual frequency mean (SE) for responders	0.14 (0.00)		Medical Data Vision Co., Ltd claims analysis^40^
Primary care visits annual frequency mean (SE) for responders	7.26 (0.04)		
Hospitalization annual frequency mean (SE) for non‐responders	0.20 (0.00)		
Primary care visits annual frequency mean (SE) for non‐responders	11.27 (0.08)		
Monitoring, number of tests in Years 1 or 2+ for abrocitinib patients			
Renal function test	Year 1: 3		Validated by clinical experts
	Year 2+: 1		
Complete blood count	Year 1: 3		
	Year 2+: 1		
Liver function test	Year 1: 3		
	Year 2+: 1		
Tuberculosis testing	Year 1: 1		
	Year 2+: 0		
Hepatitis B and C testing	Year 1: 1		
	Year 2+: 0		
Chest X‐ray	Year 1: 1		
	Year 2+: 0		
	Abrocitinib	SoC	
Annual adverse event rates, by treatment, %			
Acne	9.3	0.0	Pooled JADE trial data,^34^ converted into annual rates
Conjunctivitis	2.7	7.3	
Dermatitis atopic	9.3	11.9	
Headache	13.1	14.2	
Nasopharyngitis	27.1	20.7	
Nausea	13.1	4.9	
Oral herpes	5.4	2.5	
Upper respiratory tract infection	15.5	14.2	
Urinary tract infection	5.4	4.9	
Folliculitis	5.4	9.6	
Herpes zoster	5.4	0.0	
Utility values, by treatment and response status			
SoC, Responders	0.86		Simpson et al.^37^
SoC, Non‐responders	0.61		
Abrocitinib, Responders	0.88		
Abrocitinib, Non‐responders	0.78		
AD‐related productivity loss, percentage of hours lost at baseline, %			
Abrocitinib or SoC, mean (SE)	33.6 (6.2)		Estimation based on Murota et al.^6^
AD‐related productivity loss, treatment effect (LSM)			
Abrocitinib or SoC, Responders, mean (SE)	−23.4 (0.9)		Estimation based on Murota et al.^7^
Abrocitinib or SoC, Non‐responders, mean (SE)	0.0 (0.0)		
AD‐related productivity loss, other inputs, %			
Percent of employed Japanese adults, mean (SE)	83.30 (16.66)		Derived from Japanese government statistics^42^, ^43^
Work hours per week, mean (SE)	36.80 (7.36)		Derived from Japanese government statistics^42^, ^43^
Average hourly wage, mean (SE)	¥2353 (¥471)		Derived from Japanese government statistics^42^, ^43^
Average retirement age, mean (SE)	65.00 (13.00)		Assumption

Abbreviations: AD, atopic dermatitis; CADTH, Canada's Drug Health and Technology Agency; JADE, JAK1 Atopic Dermatitis Efficacy and Safety; LSM, least squares mean; NHI, Japanese National Health Insurance; NMA, network meta‐analysis, SE, standard error; SoC, standard of care.

### 2.6 Sensitivity analysis

One‐way sensitivity analysis (OWSA) was conducted by switching a parameter's base case value to its (estimated) 95% confidence interval (CI) lower or upper bound, informed by abrocitinib clinical trial data wherever applicable. In the absence of uncertainty data (e.g., CI or standard error), 20% of the base case mean value was assumed. Probabilistic sensitivity analysis (PSA) with 1000 iterations was also performed. In each iteration, the base case values of all parameters were simultaneously sampled using random values and their assigned statistical distributions (e.g., beta, normal, and gamma distribution).

### 2.7 Scenario analysis

In the scenario analysis, a shift in perspective from societal to payer was explored. The productivity losses were excluded in the payer perspective scenario analysis.

## 3 RESULTS

### 3.1 Base case analysis

#### 3.1.1 Deterministic results

The deterministic results for the base case from the societal perspective are presented in Table 2. Abrocitinib yielded an additional 0.75 QALYs compared with SoC, and the incremental cost was ¥2 270 386 ($17 265.6). Therefore, the ICER was ¥3 034 514 ($23 076.5) per QALY gained compared with SoC.

**TABLE 2:** Deterministic pairwise results: base case (societal perspective).

	Treatment arms	
	Abrocitinib	SoC
Total LY	46.05	46.05
Total QALY	18.86	18.11
Number of years on treatment	46.05	46.05
Total years in response	3.66	0.36
Number of years receiving subsequent treatment	42.39	45.69
Total Cost	¥16 004 964 ($121 712.6)	¥11 528 187 ($87 668.2)
Drug acquisition cost	¥9 687 198 ($73 668.0)	¥5 032 647 ($38 271.7)
AE cost	¥21 088 ($160.4)	¥19 213 ($146.1)
Health care resource use cost	¥6 287 608 ($47 815.2)	¥6 476 327 ($49 250.4)
Monitoring cost	¥9070 ($69.0)	¥0 ($0.0)
Lost income	¥22 767 378 ($173 138.6)	¥24 973 769 ($189 917.5)
Incremental QALY	0.75	
Incremental Cost	¥2 270 386 ($17 265.6)	
ICER (per QALY), base case	¥3 034 514 ($23 076.5)	

Abbreviations: AE, adverse event; ICER, incremental cost‐effectiveness ratio; LY, life year; N/R, not relevant; QALY, quality adjusted life year; SoC, standard of care.

#### 3.1.2 Sensitivity analysis

**FIGURE 2:**
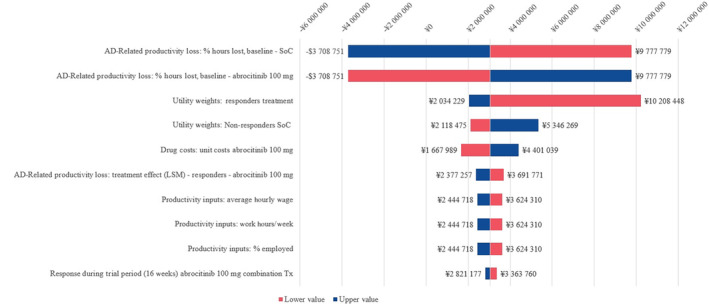
Tornado diagram of the top 10 most influential parameters for the incremental cost‐effectiveness ratio (ICER) of abrocitinib versus standard of care (SoC). Inputs were individually varied by ±20% to capture the effect on the ICER. *Note*: For the top two parameters, abrocitinib dominates SoC with positive incremental quality‐adjusted life years (QALYs) and lower costs. AD, atopic dermatitis; LSM, least squares mean; Tx, treatment.

From the base‐case societal perspective, the tornado diagram from the OWSA (Figure 2) revealed that the most influential set of parameters was the baseline proportion of working hours lost for patients with AD. This was followed by treatment‐specific, response‐based utility values (especially the abrocitinib responder utility weight and the SoC non‐responder utility weight) and the abrocitinib drug costs.

**FIGURE 3:**
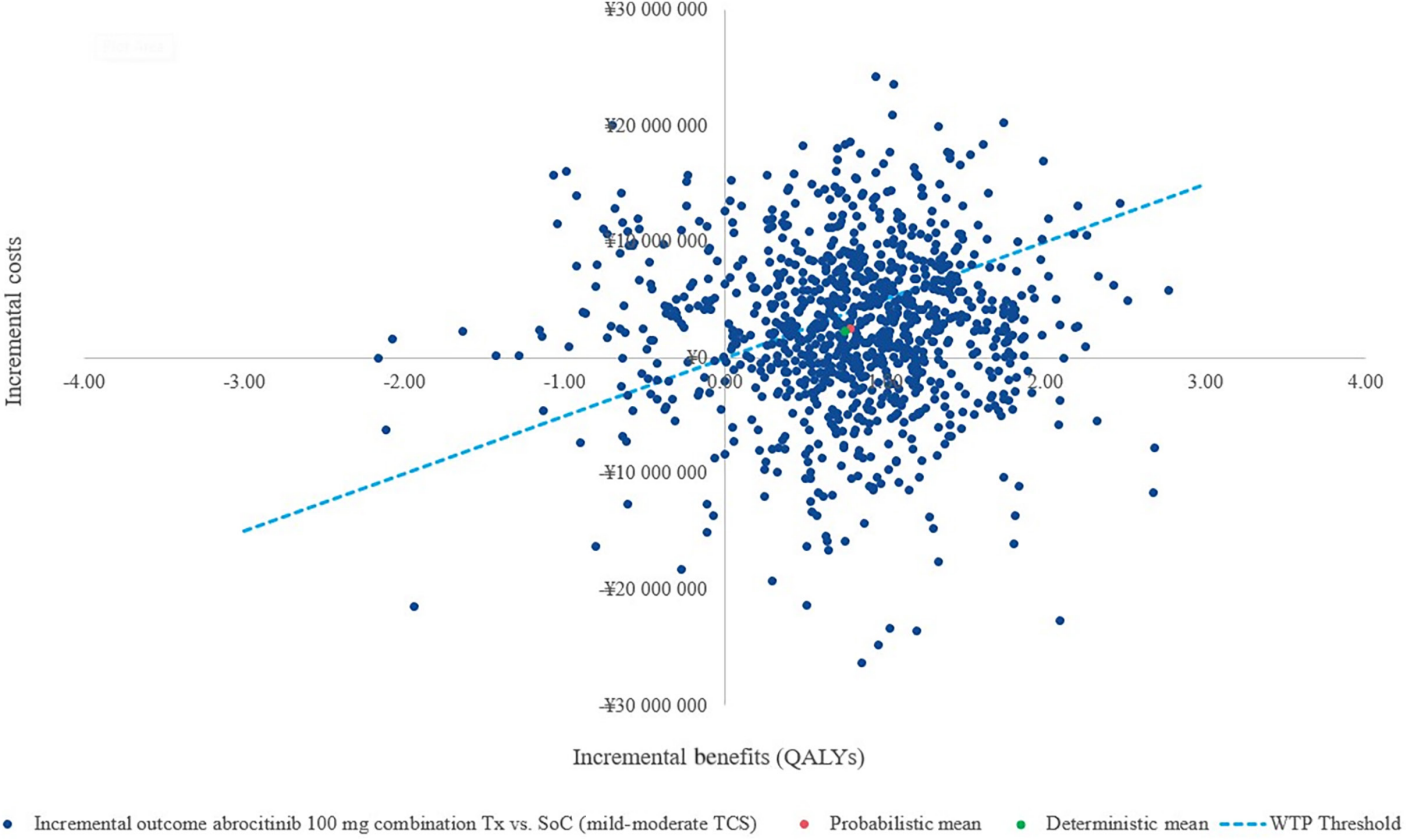
Scatter plot of 1000 probabilistic sensitivity analysis (PSA) iterations on the cost‐effectiveness (CE) plane. The dotted blue line represents a willingness‐to‐pay threshold of ¥5 000 000 ($38 023.4) per quality‐adjusted life year (QALY). Any PSA iterations under the dotted line represent cost‐effective incremental cost‐effectiveness ratio SoC, standard of care; TCS, topical corticosteroid; Tx, treatment; WTP, willingness to pay.

**FIGURE 4:**
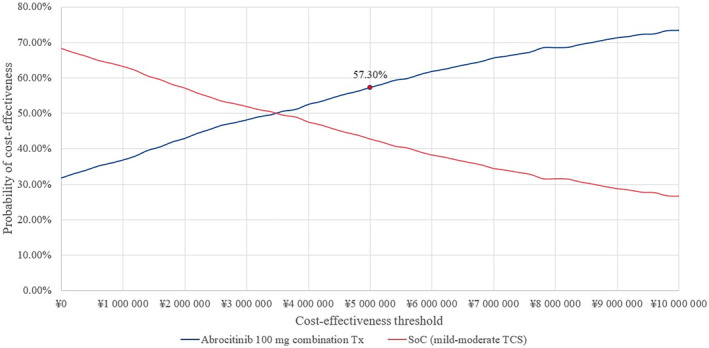
Cost‐effectiveness acceptability curve of abrocitinib versus standard of care (SoC) under a willingness‐to‐pay threshold of ¥5 000 000 ($38 023.4) per quality‐adjusted life year (QALY). TCS, topical corticosteroids; Tx, treatment.

PSA based on 1000 iterations yielded a mean ICER of ¥3 334 805 ($25 360.1) per QALY from the base‐case societal perspective. The scatter plot, presented in Figure 3 shows that the probabilistic mean incremental costs and QALYs are fairly close to the deterministic results. The cost‐effectiveness acceptability curve is presented in Figure 4, which demonstrates that at a WTP threshold of ¥5 000 000 ($38 023.4) per QALY, abrocitinib was 57.3% more likely to be cost‐effective than SoC, from the societal perspective.

### 3.2 Scenario analysis

#### 3.2.1 Deterministic results

The deterministic results for the payer perspective scenario are presented in Table 3. The shift in perspective did not affect the QALY gains; in both the payer and societal perspectives, abrocitinib yielded an additional 0.75 QALYs compared with SoC. Abrocitinib was estimated to have an incremental cost of ¥4 476 777 ($34 044.4), resulting in an ICER of ¥5 983 495 ($45 502.6) per QALY gained compared with SoC.

**TABLE 3:** Scenario analysis (payer): deterministic pairwise results.

	Treatment arms	
	Abrocitinib	SoC
Total LY	46.05	46.05
Total QALY	18.86	18.11
Number of years on treatment	46.05	46.05
Total years in response	3.66	0.36
Number of years receiving subsequent treatment	42.39	45.69
Total Cost	¥16 004 964 ($121 712.6)	¥11 528 187 ($87 668.2)
Drug acquisition cost	¥9 687 198 ($73 668.0)	¥5 032 647 ($38 271.7)
AE cost	¥21 088 ($160.4)	¥19 213 ($146.1)
Health care resource use cost	¥6 287 608	¥6 476 327
Monitoring cost	¥8175	¥0
Incremental QALY	0.75	
Incremental Cost, excluding lost income	¥4 476 777 ($34 044.4)	
ICER (¥ per QALY), scenario excluding lost income (payer perspective)	¥5 983 495 ($45 502.6)	

Abbreviations: AE, adverse event; ICER, incremental cost‐effectiveness ratio; LY, life year; QALY, quality adjusted life year; SoC, standard of care.

#### 3.2.2 Sensitivity analysis

Both OWSA and PSA based on 1000 iterations were performed under the payer perspective and the results are presented in Figures S1–S3. Based on the OWSA, the most influential parameters from a payer perspective included responder and non‐responder utility weights, abrocitinib drug costs, and trial‐based response inputs at 16 and 52 weeks (Figure S1). The PSA of 1000 iterations resulted in a mean ICER of ¥5 846 604 ($44 461.5) per QALY, which is similar to the deterministic results. Compared with the base case analysis, the range of estimated incremental costs was narrow (Figure S2). Using a WTP threshold of ¥5 000 000 ($38 023.4) per QALY, abrocitinib was 46.9% likely to be cost‐effective compared with SoC from the payer perspective (Figure S3).

## 4 DISCUSSION

Prior to this analysis, abrocitinib has demonstrated superior efficacy and safety versus SoC across a variety of Phase 3 randomized controlled clinical trials for the treatment of moderate‐to‐severe AD.^11^ As a result, abrocitinib has been approved for the treatment of moderate‐to‐severe AD in adults and adolescents aged ≥12 years with inadequate response to existing therapies in many countries, including Japan. This evidence base has been complemented by real‐world studies, demonstrating the effectiveness of abrocitinib as a treatment for moderate‐to‐severe AD in clinical practice.^45^

While the clinical benefit of abrocitinib has been assessed in detail, this analysis is the first to study its cost‐effectiveness in Japan. With an ICER of ¥3 034 514 ($23 076.5) per QALY and a WTP threshold of ¥5 000 000 ($38 023.4) per QALY, this CEA demonstrates that abrocitinib is a cost‐effective treatment option for moderate‐to‐severe AD compared with SoC from a Japanese societal perspective. The ICER for this study from the payer perspective was ¥5 983 495 ($45 502.6) per QALY gained compared with SoC, although the results may depend on the change in utility weights for responders treated with abrocitinib.

While many countries recommend that CEAs adopt a payer perspective in the base case or suggest only adopting a societal perspective as a scenario, this CEA assessed AD from a societal perspective in the base case, in line with HTA recommendations from countries such as Denmark,^46^ the Netherlands^47^ and Sweden.^48^ The use of a societal perspective in AD is critical as it allows for the explicit consideration of patient productivity loss, which has been previously proven to be significant in this indication.^6^, ^7^, ^8^ The inclusion of productivity loss is also recommended by other guidelines and publications, including the International Society for Pharmacoeconomics and Outcomes Research Value Flower framework.^49^, ^50^

A systematic literature review of economic evaluations of the treatment of AD, psoriasis, and chronic urticaria by Igarashi et al.,^51^ outlined that out of the 123 included studies, only 10.6% considered a base‐case societal perspective and 76.4% did not report costs based on productivity changes. Among the 14 studies that focused on AD, only three were conducted from a societal perspective and only four considered productivity‐related costs. One of the AD studies was based in Japan, and while productivity costs were estimated, the perspective was not explicitly stated.^6^ An additional Japanese CEA not included in the Igarashi study, assessed the cost‐effectiveness of delgocitinib, a topical JAKi for the treatment of AD,^52^ however, this paper undertook a healthcare perspective and focused on direct costs only. Consequently, the analysis outlined in this report fills a gap within the Japanese and global AD evidence base with the adoption of a societal perspective and inclusion of productivity‐related costs.

While the economic evaluation evidence base may be small for AD, CEAs on other similar skin diseases, such as psoriasis, can be used to validate this analysis. As with AD, psoriasis is not life‐threatening and has many recently approved biologic treatments.^51^ This CEA is comparable in terms of model structure and key model assumptions to CEAs on psoriasis. Specifically, a recent systematic literature review which synthesized the cost and cost‐effectiveness of treatments for psoriatic arthritis found that most CEAs, excluding the studies that used direct empirical observation, applied a hybrid decision tree and Markov model. Also, these studies often compared biologic treatments with conventional therapies, and suggested that these patients face substantial productivity losses and indirect costs.^53^

This analysis was performed in accordance with best practices and the guidelines for performing CEAs in order to conduct an economic evaluation that best reflects the actual treatment situation.^54^ While CEAs often use only available data and may be based on heterogeneous populations,^55^ this study also incorporated a number of methods to take into account and adjust for heterogeneity of some of the input parameters. The clinical effectiveness and safety inputs were based on all relevant abrocitinib Phase 3 trials, and comparative effectiveness data for abrocitinib versus SoC were derived from an NMA. Where data were not available, plausible assumptions were made and sensitivity analyses was performed to assess the uncertainty. To explicitly consider the burden that AD poses to patients' quality of life, this analysis utilized EASI‐75 with ≥4‐point improvement in the DLQI, a quality‐of‐life measure specific to dermatological diseases, as the efficacy outcome for defining responders. In addition, for the HCRU analysis, costs and productivity‐related inputs were informed by a Japanese claims analysis and a literature review. In terms of the analytical method, the CEA included PSA, extensive OWSA, and a scenario analysis to assess the sensitivity and robustness of the model systematically and comprehensively.

Despite the above, there are several limitations to consider in this analysis. The current study considered only the binary pattern of clinical response (presence or absence) rather than multiple levels of clinical response. On one hand, this may have led to further simplification of clinical practice of AD. On the other hand, consideration of multiple levels of clinical response would require acquisition of cost and utility for each level of the response, but it is difficult to obtain those values in practice.

Given the lack of long‐term effectiveness data from abrocitinib clinical trials, assumptions on treatment waning and discontinuation were made that may not accurately represent clinical practice. Utility values in the model base case were derived from published dupilumab trial values.^37^ Dupilumab utility values were deemed more appropriate as abrocitinib trials excluded patients with clinically significant depression, which substantially affected utility values. In terms of abrocitinib dosing, while both 100 mg and 200 mg were studied in the JADE program clinical trials, the current analysis only considered 100 mg dosing as it was deemed the most likely dosing for Japanese patients. The result of OWSA demonstrated that the potential impact of assumptions on treatment waning and discontinuation was likely to be less than those of other major parameters in the model because they were not among the top 10 parameters with the greatest impact on the result of cost‐effectiveness analysis.

The SoC considered in this analysis is consistent with the SoC used in the past clinical trial of abrocitinib, but it did not necessarily include the full range of the SoC that could be used in clinical practice in Japan.

Furthermore, this analysis did not include other, newly available AD treatments in Japan, such as dupilumab. In the coming years, several new and expensive systemic treatments are likely to be approved in Japan, such as lebrikizumab,^56^ tralokinumab,^57^, ^58^ nemolizumab,^59^ and tezepelumab.^60^ Consequently, future CEAs will be critical to assessing the economic value and cost‐effectiveness of abrocitinib and emerging treatments for AD to help manage the increasing expenditure on AD healthcare in Japan.

In conclusion, from a Japanese societal perspective, abrocitinib demonstrated superior QALYs and, with a WTP threshold of ¥5 000 000 ($38 023.4) per QALY, can be considered cost‐effective compared with SoC as a treatment for moderate‐to‐severe AD in adult patients with previous immunosuppressant exposure.

## CONFLICT OF INTEREST STATEMENT

The authors declare the following potential conflicts of interest with respect to the research, authorship and/or publication of this article: Akio Tanaka reports lecture fees not related to the submitted work from AbbVie GK, Eli Lilly Japan K.K., Kaken Pharmaceutical Co., Ltd., Kyorin Pharmaceutical Co., Ltd., Mitsubishi Tanabe Pharma Corporation, Pfizer Japan Inc., Sanofi K.K., Taiho Pharmaceutical Co., Torii Pharmaceutical Co., Ltd., and Maruho Co., Ltd., advisory fee from Pfizer Japan Inc., advisory fees not related to the submitted work from Eli Lilly Japan K.K., Sanofi K.K., LEO Pharma K.K., and Maruho Co., Ltd., and research grants not related to the submitted work from Eli Lilly Japan K.K., Maruho Co., Ltd., Mitsubishi Tanabe Pharma Corporation, Taiho Pharmaceutical Co., Teijin Pharma Limited, and Torii Pharmaceutical Co., Ltd. Akira Yuasa, Kazumasa Kamei, Mitsuhiro Nagano, and Kouki Nakamura are full‐time employees of Pfizer Japan Inc. Toshiaki Murofushi is a full‐time employee of INTAGE Healthcare Inc. Annika Bjerke is a full‐time employee of Lumanity. Shunya Ikeda has no conflicts of interest to declare.

## Supporting information


Figures S1–S3.

## References

[1] Weidinger S , Novak N . Atopic dermatitis. Lancet. 2016;387:1109–1122. 26377142 10.1016/S0140-6736(15)00149-X

[2] Iznardo H , Roe E , Serra‐Baldrich E , Puig L . Efficacy and safety of JAK1 inhibitor abrocitinib in atopic dermatitis. Pharmaceutics. 2023;15:385. 36839707 10.3390/pharmaceutics15020385 PMC9960033

[3] Cheng J , Wu JJ , Han G . Epidemiology and characterization of atopic dermatitis in east Asian populations: a systematic review. Dermatol Ther. 2021;11:707–717. 10.1007/s13555-021-00516-w PMC8163933 33835410

[4] Katoh N , Ohya Y , Ikeda M , Ebihara T , Katayama I , Saeki H , et al. Japanese guidelines for atopic dermatitis 2020. Allergol Int. 2020;69:356–369. 32265116 10.1016/j.alit.2020.02.006

[5] Saeki H , Ohya Y , Furuta J , Arakawa H , Ichiyama S , Katsunuma T , et al. Executive summary: Japanese guidelines for atopic dermatitis (ADGL) 2021. Allergol Int. 2022;71:448–458. 36064654 10.1016/j.alit.2022.06.009

[6] Murota H , Inoue S , Yoshida K , Ishimoto A . Cost of illness study for adult atopic dermatitis in Japan: a cross‐sectional web‐based survey. J Dermatol. 2020;47:689–698. 32383191 10.1111/1346-8138.15366 PMC7384180

[7] Arima K , Gupta S , Gadkari A , Hiragun T , Kono T , Katayama I , et al. Burden of atopic dermatitis in Japanese adults: analysis of data from the 2013 National Health and Wellness Survey. J Dermatol. 2018;45:390–396. 29388334 10.1111/1346-8138.14218 PMC5947641

[8] Kamei K , Hirose T , Yoshii N , Tanaka A . Burden of illness, medication adherence, and unmet medical needs in Japanese patients with atopic dermatitis: a retrospective analysis of a cross‐sectional questionnaire survey. J Dermatol. 2021;48:1491–1498. 34231235 10.1111/1346-8138.16054 PMC9291885

[9] Regeneron Pharmaceuticals Inc . Regeneron announces approval of dupixent® (dupilumab) in japan for the treatment of atopic dermatitis. 2018. Available from: https://newsroom.regeneron.com/news‐releases/news‐release‐details/regeneron‐announces‐approval‐dupixentr‐dupilumab‐japan‐treatment/

[10] Kamata M , Tada Y . A literature review of real‐world effectiveness and safety of Dupilumab for atopic dermatitis. JID Innov. 2021;1:100042. 34909737 10.1016/j.xjidi.2021.100042 PMC8659403

[11] Saeki H , Akiyama M , Abe M , Igarashi A , Imafuku S , Ohya Y , et al. English version of Japanese guidance for the use of oral Janus kinase (JAK) inhibitors in the treatments of atopic dermatitis. J Dermatol. 2023;50:e1–e19. 36412059 10.1111/1346-8138.16635

[12] Pfizer Inc . Japan's MHLW approves Pfizer's CIBINQO® (abrocitinib) for adults and adolescents with moderate to severe atopic dermatitis. 2021. Available from: https://www.pfizer.com/news/press‐release/press‐release‐detail/japans‐mhlw‐approves‐pfizers‐cibinqor‐abrocitinib‐adults

[13] Pfizer Japan Inc . CIBINQO (abrocitinib), Package Inserts. 2021 [updated July, 2023]. Available from: https://www.pmda.go.jp/PmdaSearch/iyakuDetail/ResultDataSetPDF/672212_4490037F1026_1_04

[14] Nobbe S , Dziunycz P , Mühleisen B , Bilsborough J , Dillon SR , French LE , et al. IL‐31 expression by inflammatory cells is preferentially elevated in atopic dermatitis. Acta Derm Venereol. 2012;92:24–28. 22041865 10.2340/00015555-1191

[15] Yang X , Zheng SG . Interleukin‐22: a likely target for treatment of autoimmune diseases. Autoimmun Rev. 2014;13:615–620. 24418299 10.1016/j.autrev.2013.11.008 PMC3966954

[16] Brunner PM , Guttman‐Yassky E , Leung DY . The immunology of atopic dermatitis and its reversibility with broad‐spectrum and targeted therapies. J Allergy Clin Immunol. 2017;139(4S):S65–S76. 28390479 10.1016/j.jaci.2017.01.011 PMC5405702

[17] Ministry of Health Labour and Welfare . Guidelines for promoting the optimal use of baricitinib (Brand name: 2‐mg Olumiant tablets, 4‐mg Olumiant tablets) Atopic dermatitis. 2020 [updated March, 2024]. Available from: https://www.pmda.go.jp/files/000267565.pdf

[18] Ministry of Health Labour and Welfare . Guidelines for promoting the optimal use of upadacitinib hydrate (brand name: 7.5‐mg Rinvoq tablets, 15‐mg Rinvoq tablets, 30‐mg Rinvoq tablets) Atopic dermatitis. 2021. Available from: https://www.pmda.go.jp/files/000243653.pdf

[19] Ministry of Health Labour and Welfare . Guidelines for promoting the optimal use of abrocitinib (Brand name: 200‐mg Cibinqo tablets, 100‐mg Cibinqo tablets, 50‐mg Cibinqo tablets) Atopic dermatitis. 2022. Available from: https://www.pmda.go.jp/files/000249916.pdf

[20] Silverberg JI , Thyssen JP , Simpson EL , Yosipovitch G , Stander S , Valdez H , et al. Impact of Oral Abrocitinib monotherapy on patient‐reported symptoms and quality of life in adolescents and adults with moderate‐to‐severe atopic dermatitis: a pooled analysis of patient‐reported outcomes. Am J Clin Dermatol. 2021;22:541–554. 33954933 10.1007/s40257-021-00604-9 PMC8200343

[21] Canadian Agency for Drugs and Technologies in Health . Reimbursement review abrocitinib (Cibinqo). 2023. Available from: https://www.cadth.ca/sites/default/files/DRR/2023/SR0686‐Cibinqo.pdf 38498644

[22] Institute for Clinical and Economic Review . JAK inhibitors and monoclonal antibodies for the treatment of atopic dermatitis: effectiveness and value: Final evidence report. 2023. Available from: https://icer.org/wp‐content/uploads/2023/02/Atopic‐Dermatitis_Final‐Evidence‐Report_Unmasked_02272023.pdf 10.18553/jmcp.2022.28.1.108 PMC10373035 34949111

[23] National Institute for Health and Care Excellence . [TA534] Dupilumab for treating moderate to severe atopic dermatitis. 2018. Available from: https://www.nice.org.uk/guidance/TA534 41406311

[24] National Institute for Health and Care Excellence . [TA681] Baricitinib for treating moderate to severe atopic dermatitis. 2021. Available from: https://www.nice.org.uk/guidance/TA681 40737470

[25] National Institute for Health and Care Excellence . [TA814] Abrocitinib, tralokinumab or upadacitinib for treating moderate to severe atopic dermatitis – Committee Papers. 2022. Available from: https://www.nice.org.uk/guidance/ta814/documents/committee‐papers 40504959

[26] Scottish Medicines Consortium . Dupilumab (Dupixent). 2018. Available from: https://www.scottishmedicines.org.uk/medicines‐advice/dupilumab‐dupixent‐fullsubmission‐smc2011/

[27] Center for Outcomes Research and Economic Evaluation for Health National Institute of Public Health (C2H) . Guideline for preparing cost‐effectiveness evaluation to the Central Social Insurance Medical Council. 2022. Available from: https://c2h.niph.go.jp/tools/guideline/guideline_en.pdf

[28] Drost R , van der Putten IM , Ruwaard D , Evers S , Paulus ATG . Conceptualizations of the societal perspective within economic evaluations: a systematic review. Int J Technol Assess Health Care. 2017;33:251–260. 28641592 10.1017/S0266462317000526

[29] Canadian Agency for Drugs and Technologies in Health . Pharmacoeconomic report for Dupixient SR0636. 2020. Available from: https://www.cadth.ca/sites/default/files/cdr/pharmacoeconomic/sr0636‐dupixent‐pharmacoeconomic‐review‐report.pdf

[30] Rudmik L , Drummond M . Health economic evaluation: important principles and methodology. Laryngoscope. 2013;123:1341–1347. 23483522 10.1002/lary.23943

[31] Hasegawa M , Komoto S , Shiroiwa T , Fukuda T . Formal implementation of cost‐effectiveness evaluations in Japan: a unique health technology assessment system. Value Health. 2020;23:43–51. 31952673 10.1016/j.jval.2019.10.005

[32] Silverberg JI , Thyssen JP , Fahrbach K , Mickle K , Cappelleri JC , Romero W , et al. Comparative efficacy and safety of systemic therapies used in moderate‐to‐severe atopic dermatitis: a systematic literature review and network meta‐analysis. J Eur Acad Dermatol Venereol. 2021;35:1797–1810. 33991374 10.1111/jdv.17351 PMC8453983

[33] Bieber T , Simpson EL , Silverberg JI , Thaçi D , Paul C , Pink AE , et al. Abrocitinib versus Placebo or Dupilumab for atopic dermatitis. N Engl J Med. 2021;384:1101–1112. 33761207 10.1056/NEJMoa2019380

[34] Pfizer Inc . Data on file. 2022.

[35] National Institute of Population and Social Security Research . All Japan: Life Table Data Series. 2022. Available from: https://www.ipss.go.jp/p‐toukei/JMD/00/index‐en.html

[36] Winthrop KL . The emerging safety profile of JAK inhibitors in rheumatic disease. Nat Rev Rheumatol. 2017;13:234–243. 28250461 10.1038/nrrheum.2017.23

[37] Simpson EL . Dupilumab improves general health‐related quality‐of‐life in patients with moderate‐to‐severe atopic dermatitis: pooled results from two randomized, controlled phase 3 clinical trials. Dermatol Ther. 2017;7:243–248. 10.1007/s13555-017-0181-6 PMC5453923 28503712

[38] Ministry of Health Labour and Welfare . National Health Insurance Tarrif (revised in 2022). 2022. Available from: https://www.mhlw.go.jp/content/12404000/000907834.pdf

[39] Organisation for Economic Co‐operation and Development . Exchange rates (yearly). 2022. Available from: https://data.oecd.org/conversion/exchange‐rates.htm

[40] Medical Data Vision . Website. 2023. Available from: https://en.mdv.co.jp/

[41] Ministry of Health Labour and Welfare . National Health Insurance Drug Price List (revised in 2024). 2024. Available from: https://www.mhlw.go.jp/content/12404000/001218724.pdf

[42] Statistics Bureau of Japan . Patient Survey 2020. 2020. Available from: https://www.e‐stat.go.jp/stat‐search/files?page=1&layout=datalist&toukei=00450022&tstat=000001031167&cycle=7&tclass1=000001166809&tclass2=000001166811&tclass3=000001166812&tclass4=000001166813&tclass5val=0

[43] Ministry of Health Labour and Welfare . Labour Force Survey 2021. 2021. Available from: https://www.stat.go.jp/data/roudou/2.html

[44] National Tax Agency . Statistical Survey of Actual Status for Salary in the Private Sector 2021. 2021. Available from: https://www.nta.go.jp/publication/statistics/kokuzeicho/minkan2021/minkan.htm

[45] Olydam JI , Schlosser AR , Custurone P , Nijsten TEC , Hijnen D . Real‐world effectiveness of abrocitinib treatment in patients with difficult‐to‐treat atopic dermatitis. J Eur Acad Dermatol Venereol. 2023;37:2537–2542. 37478296 10.1111/jdv.19378

[46] Medicinrådet . The Danish Medicines Council methods guide for assessing new pharmaceuticals. 2021. Available from: https://medicinraadet.dk/media/wq0dxny2/the_danish_medicines_council_methods_guide_for_assessing_new_pharmaceuticals_version_1‐2_adlegacy.pdf

[47] Zorginstituut Nederland . Guideline for conducting economic evaluations in healthcare [in Dutch: Richtlijn voor het uitvoeren van economische evaluaties in de gezondheidszorg]. 2016. Available from: https://www.zorginstituutnederland.nl/binaries/zinl/documenten/publicatie/2016/02/29/richtlijn‐voor‐het‐uitvoeren‐van‐economische‐evaluaties‐in‐de‐gezondheidszorg/richtlijn‐voor‐het‐uitvoeren‐van‐economische‐evaluaties‐in‐de‐gezondheidszorg.pdf

[48] Dental and Pharmaceutical Benefits Agency . Allmanna rad: Om ekonomiska utvarderingar. 2017. Available from: https://www.tlv.se/download/18.12550ff716050753615170a7/1513253615658/internationell_prisjamforelse_av_lakemedel_2017.pdf

[49] Lakdawalla DN , Doshi JA , Garrison LP Jr , Phelps CE , Basu A , Danzon PM . Defining elements of value in health care‐a health economics approach: an ISPOR Special Task Force Report [3]. Value Health. 2018;21:131–139. 29477390 10.1016/j.jval.2017.12.007

[50] Krol M , Brouwer W . How to estimate productivity costs in economic evaluations. Pharmacoeconomics. 2014;32:335–344. 24504850 10.1007/s40273-014-0132-3

[51] Igarashi A , Yuasa A , Yonemoto N , Kamei K , LoPresti M , Murofushi T , et al. A systematic literature review of economic evaluations and cost studies of the treatment of psoriasis, atopic dermatitis, and chronic urticaria. Dermatol Ther. 2022;12:1729–1751. 10.1007/s13555-022-00774-2 PMC9357586 35909186

[52] Takenaka M , Matsumoto M , Murota H , Inoue S , Shibahara H , Yoshida K , et al. Cost‐effectiveness analysis of delgocitinib in adult patients with atopic dermatitis in Japan. J Cutan Immunol Allergy. 2021;4:100–108.

[53] D'Angiolella LS , Cortesi PA , Lafranconi A , Micale M , Mangano S , Cesana G , et al. Cost and cost effectiveness of treatments for psoriatic arthritis: a systematic literature review. Pharmacoeconomics. 2018;36:567–589. 29441473 10.1007/s40273-018-0618-5

[54] Canadian Agency for Drugs and Technologies in Health . Guidelines for the economic evaluation of health technologies: Canada. 2017. Available from: https://www.cadth.ca/sites/default/files/pdf/guidelines_for_the_economic_evaluation_of_health_technologies_canada_4th_ed.pdf

[55] Sanders GD , Maciejewski ML , Basu A . Overview of cost‐effectiveness analysis. JAMA. 2019;321:1400–1401. 30855638 10.1001/jama.2019.1265

[56] Silverberg JI , Guttman‐Yassky E , Thaci D , Irvine AD , Stein Gold L , Blauvelt A , et al. Two phase 3 trials of Lebrikizumab for moderate‐to‐severe atopic dermatitis. N Engl J Med. 2023;388:1080–1091. 36920778 10.1056/NEJMoa2206714

[57] Wollenberg A , Blauvelt A , Guttman‐Yassky E , Worm M , Lynde C , Lacour JP , et al. Tralokinumab for moderate‐to‐severe atopic dermatitis: results from two 52‐week, randomized, double‐blind, multicentre, placebo‐controlled phase III trials (ECZTRA 1 and ECZTRA 2). Br J Dermatol. 2021;184:437–449. 33000465 10.1111/bjd.19574 PMC7986411

[58] Blauvelt A , Langley RG , Lacour JP , Toth D , Laquer V , Beissert S , et al. Long‐term 2‐year safety and efficacy of tralokinumab in adults with moderate‐to‐severe atopic dermatitis: interim analysis of the ECZTEND open‐label extension trial. J Am Acad Dermatol. 2022;87:815–824. 35863467 10.1016/j.jaad.2022.07.019

[59] Kabashima K , Matsumura T , Komazaki H , Kawashima M , Nemolizumab JPSG . Trial of Nemolizumab and topical agents for atopic dermatitis with pruritus. N Engl J Med. 2020;383:141–150. 32640132 10.1056/NEJMoa1917006

[60] Simpson EL , Parnes JR , She D , Crouch S , Rees W , Mo M , et al. Tezepelumab, an anti‐thymic stromal lymphopoietin monoclonal antibody, in the treatment of moderate to severe atopic dermatitis: a randomized phase 2a clinical trial. J Am Acad Dermatol. 2019;80:1013–1021. 30550828 10.1016/j.jaad.2018.11.059

