# Supplementary material for: Cost‐effectiveness analysis of abrocitinib compared with standard of care in adult moderate‐to‐severe atopic dermatitis in Japan
Source: J Dermatol. 2024 Apr 22;51(6):759–71. doi: 10.1111/1346-8138.17234 (PMC11484149; doi:10.1111/1346-8138.17234)
Supplement: Supplementary file 1 — Figures S1–S3. [file JDE-51--s001.pdf]

Cost-effectiveness analysis of abrocitinib compared with standard of care in adult moderate-to-severe atopic dermatitis in Japan.

Author names and affiliations

Akio Tanaka[1], Akira Yuasa[2], Kazumasa Kamei[2], Mitsuhiro Nagano[2], Toshiaki Murofushi[3], Annika Bjerke[4], Kouki Nakamura[2], Shunya Ikeda[5]

[1]Department of Dermatology, Graduate School of Biomedical and Health Sciences, Hiroshima University, Hiroshima, Japan

[2]Pfizer Japan Inc., Tokyo, Japan

[3]INTAGE Healthcare Inc., Tokyo, Japan

[4]Lumanity, Bethesda, Maryland, United States

[5]Department of Public Health, School of Medicine, International University of Health and Welfare, Narita, Japan

Corresponding authors

Name: Akira Yuasa

Address: Pfizer Japan Inc., Shinjuku Bunka Quint Building, 3-22-7, Yoyogi, Shibuya-ku, Tokyo 151-8589, Japan

Email: akira.yuasa@pfizer.com

Name: Akio Tanaka

Address: Department of Dermatology, Graduate School of Biomedical and Health Sciences, Hiroshima University, 1-2-3, Kasumi, Minami-ku, Hiroshima 734-8551, Japan.

Email: tantanakiotan@yahoo.co.jp

Supporting information - Supplementary figures -

Supplementary Figure 1. Tornado diagram of the top 10 most influential parameters for the ICER of abrocitinib vs. SoC under a payer perspective scenario

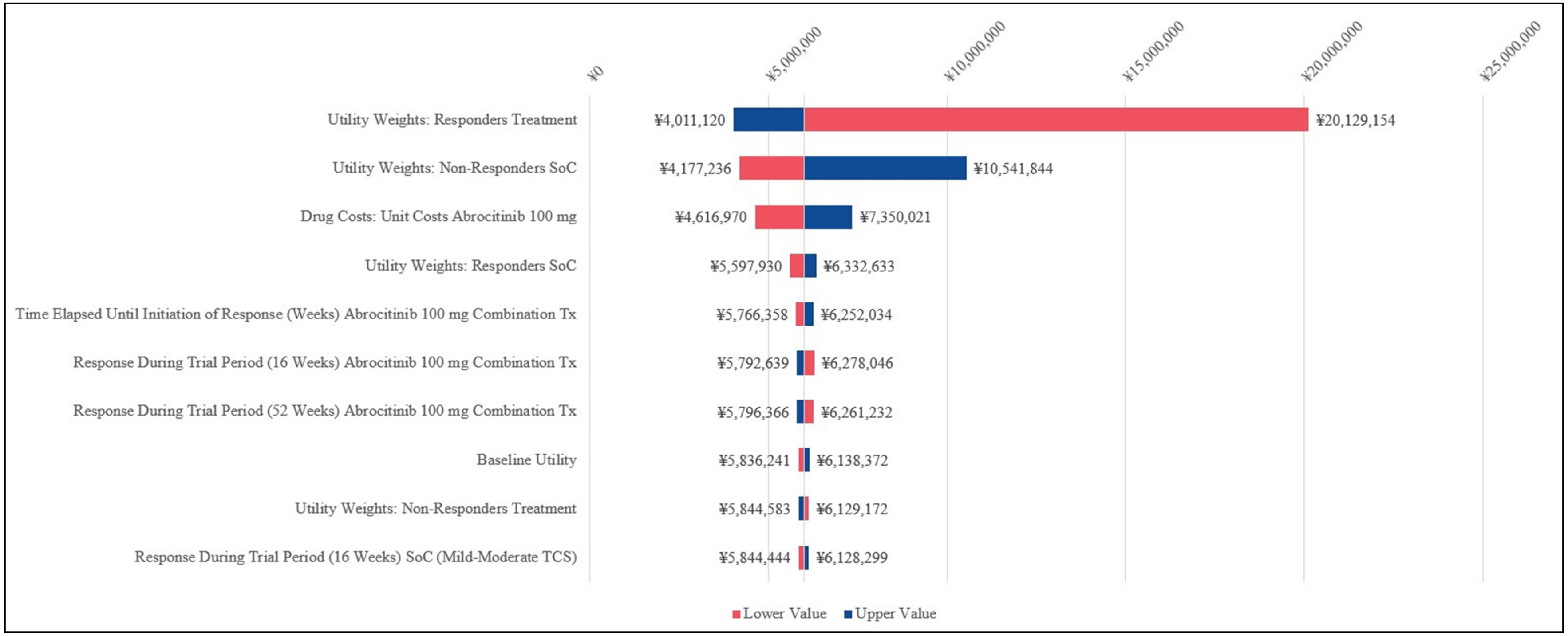

Abbreviations: ICER, incremental cost-effectiveness ratio; LSM, least squares mean; SoC, standard of care; TCS, topical corticosteroid.

Supplementary Figure 2. Scatter plot of 1,000 PSA iterations on CE plane under a payer perspective scenario

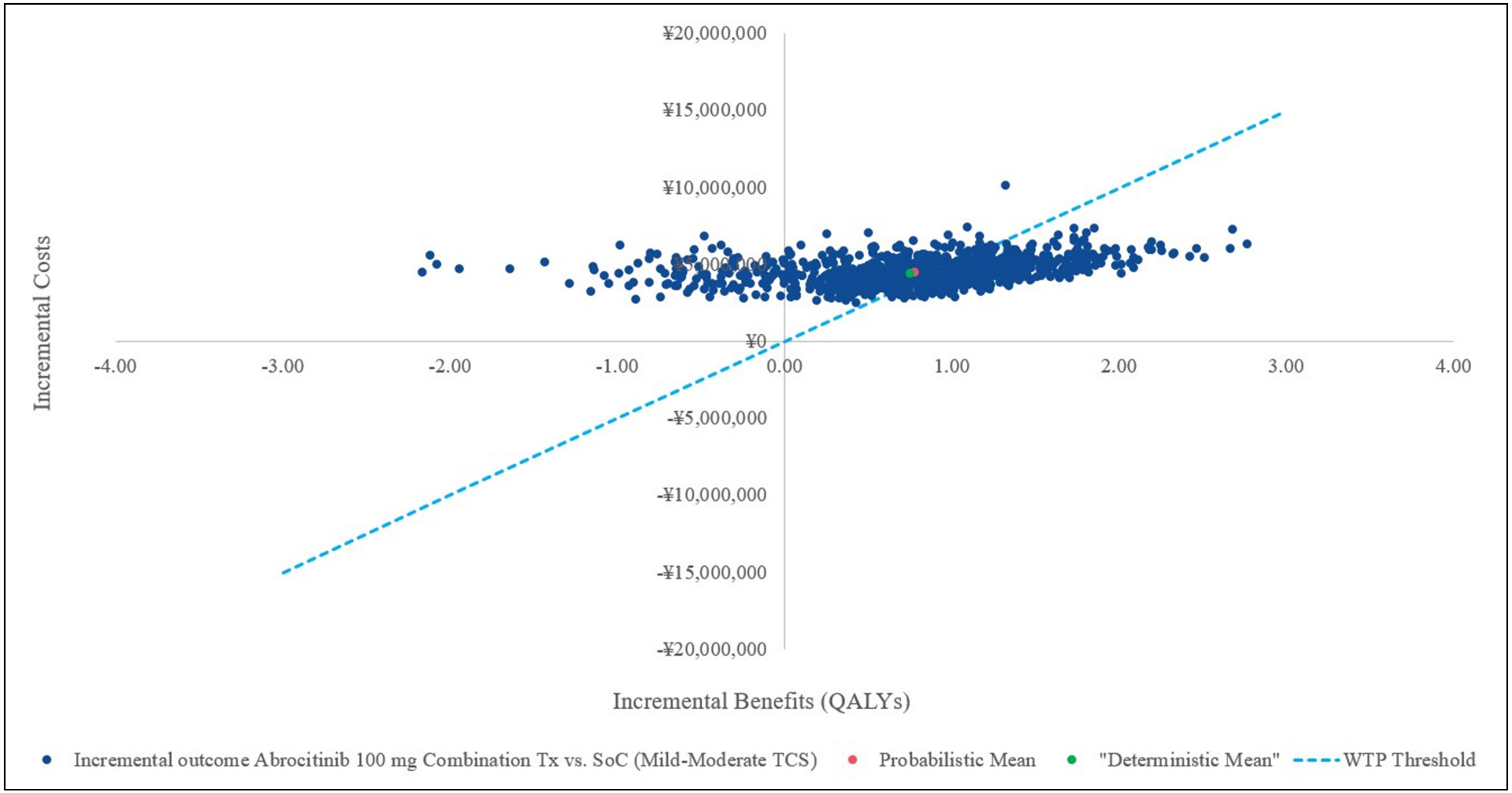

Abbreviations: CE, cost-effectiveness, PSA, probabilistic sensitivity analysis; QALYs, quality-adjusted life years; SoC, standard of care; TCS, topical corticosteroid; WTP, willingness to pay.

Supplementary Figure 3. Cost-effectiveness acceptability curve of abrocitinib vs. SoC under a payer perspective scenario

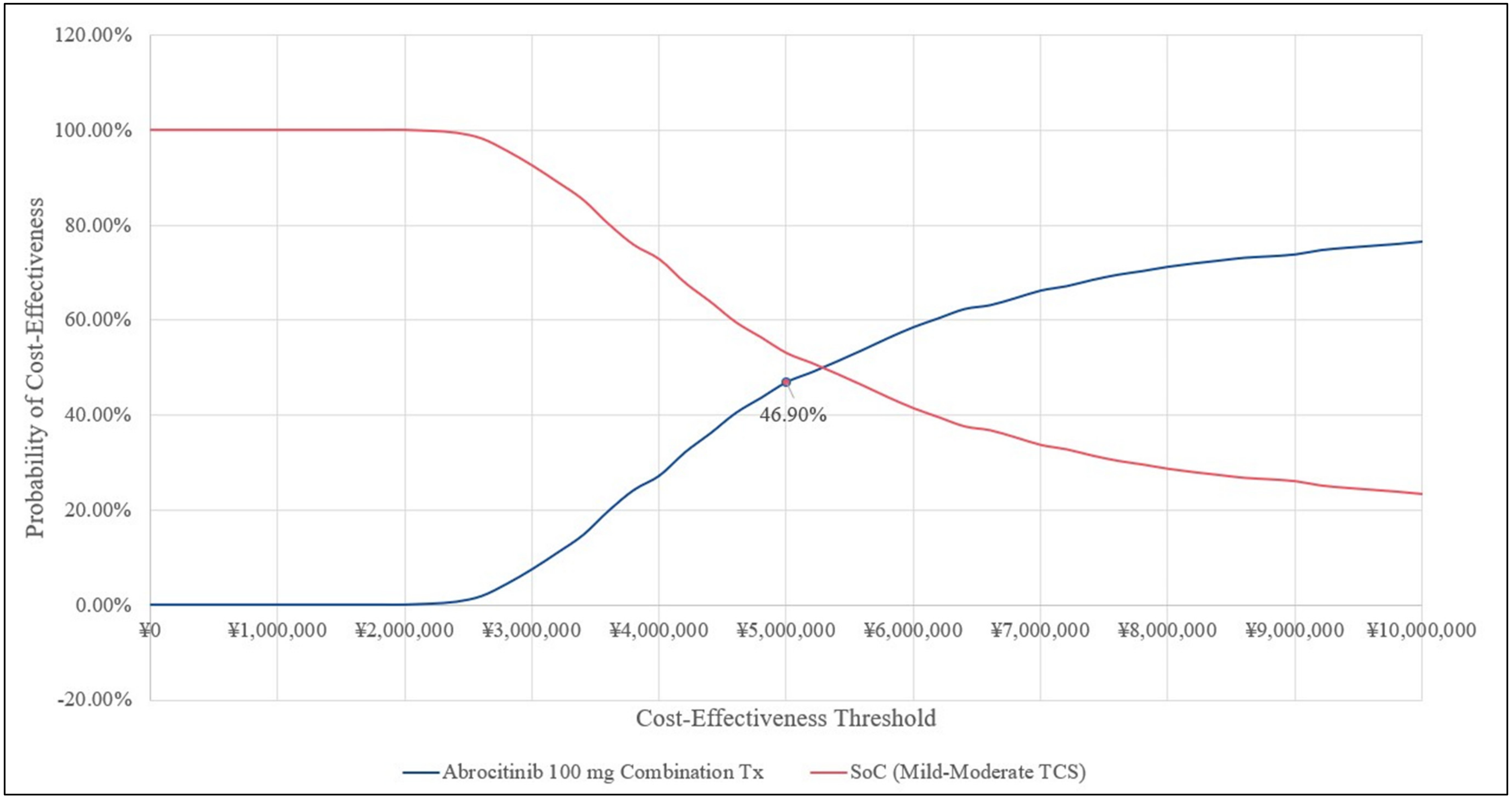

Abbreviations: SoC, standard of care; TCS, topical corticosteroid.
